# GPR84 inhibition attenuates sepsis-associated acute lung injury and preserves pulmonary endothelial barrier function associated with YY1 suppression

**DOI:** 10.3389/fphar.2026.1877785

**Published:** 2026-10-28

**Authors:** Mingzhu Wang, Huifen Feng, Chao Qin, Yujie Yin, Xuelian Huang

**Affiliations:** 1 Department of Critical Care Medicine, The Fifth Affiliated Hospital of Zhengzhou University, Zhengzhou, Henan, China; 2 Department of Infectious Diseases, The Fifth Affiliated Hospital of Zhengzhou University, Zhengzhou, Henan, China

**Keywords:** endothelial permeability, GPR84, occludin, sepsis-associated acute lung injury, YY1

## Abstract

**Introduction:**

Dysregulation of the pulmonary endothelial barrier precipitates the edema and inflammation central to sepsis-associated acute lung injury (ALI). Although the G-protein-coupled receptor GPR84 orchestrates various inflammatory responses, its specific contribution to maintaining microvascular integrity during sepsis remains largely undefined.

**Methods:**

We explored the therapeutic efficacy of GPR84 inhibition using a cecal ligation and puncture (CLP) mouse model and LPS-challenged lung microvascular endothelial cells (MLMECs).

**Results:**

Pulmonary GPR84 expression surged following septic insult, and its pharmacological blockade via selective inhibitors (GLPG1205 or TUG-2181) effectively mitigated alveolar damage. This intervention restored respiratory mechanics—including peak expiratory flow (PEF) and airway resistance (Raw)—and decreased pro-inflammatory cytokine concentrations (TNF-α, IL-6, CXCL15) within the bronchoalveolar lavage fluid. Furthermore, GPR84 inhibition curtailed vascular protein leakage and preserved Occludin expression across both experimental models. At the molecular level, GPR84 signaling appeared to facilitate the LPS-induced upregulation of the transcription factor YY1. In cultured endothelial cells, the barrier-protective effect of GPR84 inhibition was diminished by YY1 overexpression, indicating a functional role for YY1 in this in vitro system.

**Discussion:**

Collectively, these results suggest that GPR84 inhibition attenuates sepsis-associated ALI in association with YY1 suppression in vivo, identifying GPR84 as a candidate therapeutic target warranting further preclinical and clinical evaluation.

## Graphical Abstract

**Figure:**
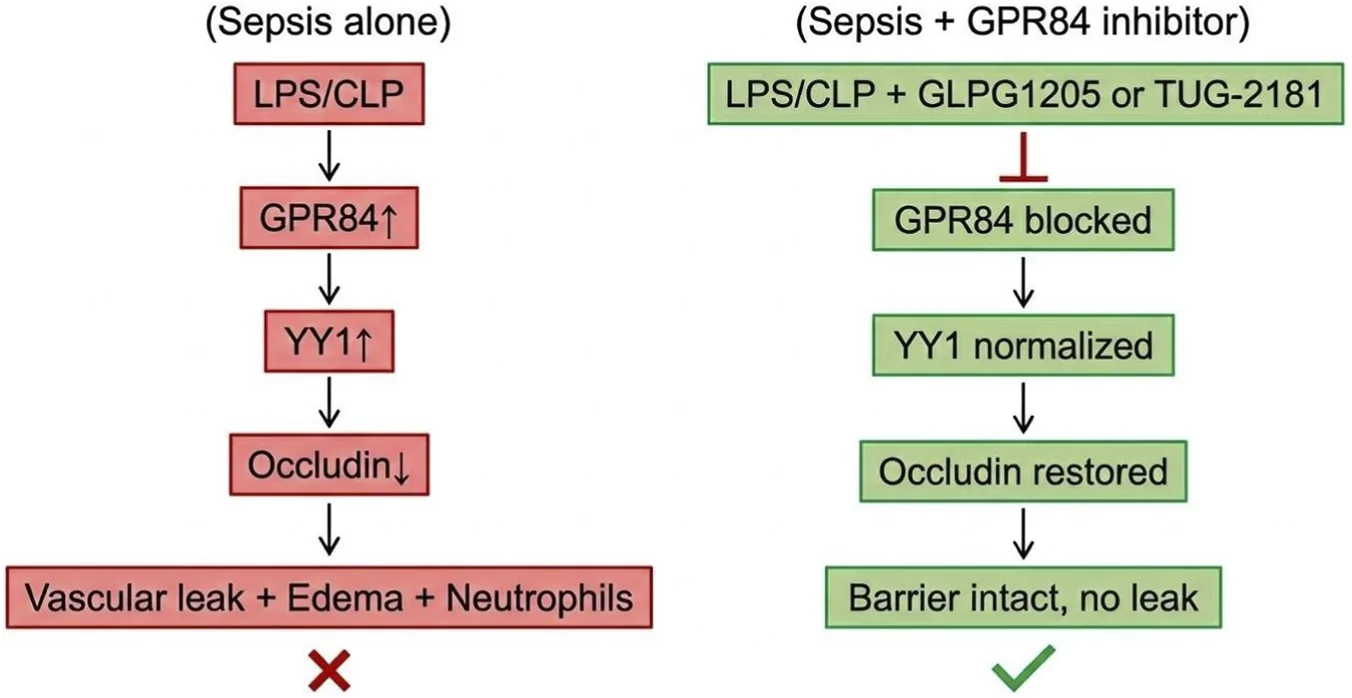

## Introduction

Sepsis remains a primary driver of global mortality, with the Global Burden of Disease framework estimating that this syndrome accounts for nearly 20% of all worldwide deaths (Rudd et al., 2020). Within this clinical landscape, the respiratory system is disproportionately affected; approximately 25%–50% of septic patients progress to acute lung injury (ALI), a complication carrying a mortality rate near 40% (Sun et al., 2023; Cheng et al., 2024). Because current management relies almost exclusively on supportive measures like protective mechanical ventilation rather than targeted pharmacology, there is a critical need to elucidate the molecular drivers of pulmonary vascular leakage (Qiao et al., 2024).

The fundamental pathology of sepsis-related ALI involves a breakdown in endothelial barrier integrity within the lung microvasculature. During septic insult, endothelial cells destabilize their intercellular bridges, which triggers the extravasation of protein-heavy fluid into the alveolar spaces and interstitium (Qiao et al., 2024; Wang et al., 2025). Research suggests this failure stems from the depletion of tight junction proteins, specifically Occludin (Dos Santos et al., 2022). This tetraspan protein is essential for maintaining paracellular seals; indeed, lung tissue from deceased septic patients shows a marked loss of Occludin (Dos Santos et al., 2022). Experimental evidence further confirms that recovering Occludin levels can fortify endothelial layers and reduce pulmonary edema (Dos Santos et al., 2022).

Transcriptional control of lung endothelial Occludin is governed by the zinc-finger protein Yin Yang 1 (YY1). Sade et al. established that YY1 binds a distal region of the *Ocln* promoter to suppress its expression in the lung, a mechanism notably absent in the brain endothelium (Sade et al., 2009). As a versatile transcription factor known for both activating and silencing genes, YY1 has gained recognition for its influence on vascular disease and development (Yang et al., 2019; Meo and de Nigris, 2024). Consequently, pathways that alter YY1 levels or its DNA-binding efficiency may serve as critical upstream regulators of vascular sealing during sepsis.

GPR84 has recently emerged as a pivotal pro-inflammatory sensor within the rhodopsin-like class A G-protein-coupled receptor family (Bai et al., 2025; Luscombe et al., 2020). Predominantly expressed in myeloid lineages, GPR84 is also present at lower concentrations in specific endothelial subsets (Marsango and Milligan, 2024; Aktar et al., 2024). A defining characteristic of GPR84 is its rapid up-regulation—in both rodent and human tissues—following exposure to pro-inflammatory stimuli or lipopolysaccharide (LPS) (Luscombe et al., 2020; Marsango et al., 2022). Once activated, GPR84 drives neutrophil functions, including ROS generation and chemotaxis, through Gαi/o-mediated pathways involving ERK1/2 and AKT kinases (Marsango and Milligan, 2024; Forsman et al., 2024). The receptor also utilizes β-arrestin recruitment to facilitate its own internalization (Marsango and Milligan, 2024).

New data explicitly link GPR84 to acute lung inflammation. In murine models of LPS-triggered injury, GPR84 expression surges; conversely, inhibiting the receptor, either through genetic knockout or chemical inhibition, attenuates lung damage and neutrophil infiltration (Wang SW. et al., 2023). Advances in medicinal chemistry have yielded sophisticated inhibitors, such as GLPG1205 (a negative allosteric modulator currently in clinical trials) and the high-potency inverse agonist TUG-2181 (Desrivot et al., 2021; Strambu et al., 2023; Kalita et al., 2023; Ieremias et al., 2025). These distinct chemical probes offer a powerful means of interrogating receptor dynamics in systemic disease.

However, the specific impact of GPR84 on the septic pulmonary endothelium remains largely unexplored, as most research focuses on myeloid cell oxidative bursts. While GPR84 is present in lung vascular cells and its inhibition can lower inflammatory markers (Gao et al., 2025), it is unknown whether its activation directly triggers tight junction loss or paracellular leakage during polymicrobial sepsis. Furthermore, the potential link between GPR84 and the Occludin-repressor YY1 has not been investigated.

In this study, we hypothesized that GPR84 induction during sepsis couples to YY1-mediated Occludin suppression, thereby compromising the alveolar-capillary barrier. We utilized the cecal ligation and puncture (CLP) model to simulate human sepsis alongside primary lung microvascular endothelial cultures (Mishra and Choudhury, 2018). By employing GLPG1205 and TUG-2181, we characterized GPR84’s contribution to barrier failure. Our results indicate that GPR84 inhibition attenuates vascular leakage and is associated with Occludin preservation and YY1 suppression, identifying GPR84 as a candidate therapeutic target for preserving vascular integrity in sepsis-associated ALI.

## Materials and methods

### Human sepsis transcriptome validation

We queried GEO via GEO2R, a web-based limma implementation requiring no local installation. Dataset GSE13904 contains 227 sample entries from 139 unique pediatric patients, with Day 1 and Day 3 longitudinal sampling. The breakdown comprises healthy controls (n = 18), SIRS (n = 22), sepsis (n = 32), and septic shock (n = 67). To mirror CLP severity and eliminate pseudo-replication, we restricted analysis to Day 1 shock (n = 67) *versus* Day 1 controls (n = 18), extracting GPR84 (probe 223,767_at) statistics directly from GEO2R output.

### Animal model of polymicrobial sepsis

C57BL/6J male mice (8–12 weeks, 22–26 g) were maintained under specific-pathogen-free conditions (12-h light/dark cycle, 22 °C ± 1 °C) with unrestricted chow and water. All animal experiments complied with the ARRIVE guidelines and were carried out in accordance with the National Institutes of Health guide for the care and use of Laboratory animals (NIH Publications No. 8023, revised 1978). The Animal Care Committee of the Fifth Affiliated Hospital of Zhengzhou University approved all procedures. For CLP-induced polymicrobial sepsis, we anesthetized mice with 2%–3% isoflurane in oxygen, performed a 1–2 cm midline laparotomy, ligated the cecum just distal to the ileocecal valve with 4–0 silk, punctured it once through-and-through with a 21-gauge needle, gently extruded a small fecal pellet, returned the cecum to the peritoneal cavity, and closed the abdominal wall and skin in layers. Sham controls underwent laparotomy without ligation or puncture. Postoperatively, every mouse received 1 mL warmed saline (s.c.) immediately and buprenorphine (0.05–0.1 mg/kg, s.c.) every 8–12 h for 48 h. In the CLP group, 2 of 10 animals (20%) died before the 24-h endpoint. These animals were excluded from endpoint analyses due to missing data, leaving 8 animals in the CLP group. The sham and inhibitor-treated groups began with n = 8 animals per group. No deaths occurred in these groups, and all 8 animals per group were included in the final analyses.

### GPR84 inhibitor treatment

We employed GLPG1205 (a negative allosteric modulator; CAS 1445847–37–9; MedChemExpress HY-135303) and TUG-2181 (an inverse agonist; IC_50_ 34 nM; CAS 2653994–24–0; MedChemExpress HY-176039). For *in vivo* experiments, septic mice received vehicle (0.5% methylcellulose), GLPG1205 (10 mg/kg), or TUG-2181 (20 mg/kg) via oral gavage once daily beginning 2 h post-CLP and continuing until the 24-h or 48-h endpoint. Dose selection was based on (i) published human pharmacokinetic data showing GLPG1205 has favorable oral bioavailability and a half-life supporting once-daily administration (Desrivot et al.); (ii) preliminary dose-finding experiments in our laboratory (data not shown) identifying 10 mg/kg GLPG1205 and 20 mg/kg TUG-2181 as the minimally effective doses that produced maximal barrier protection without observable toxicity; and (iii) the potent *in vitro* activity of TUG-2181 (IC_50_ 34 nM) (Ieremias et al., 2025), which informed dose selection based on allometric scaling and prior laboratory experience with GPR84 inhibitors. For cell studies, GLPG1205 (10 μM) and TUG-2181 (100 nM) were prepared as DMSO stocks; control wells received DMSO at ≤0.1% final concentration.

### Cell culture and LPS stimulation

We isolated primary MLMECs from adult C57BL/6J lungs by immunomagnetic CD31^+^ selection and expanded them in Endothelial Cell Growth Medium MV2 (PromoCell) with 10% FBS, 100 U/mL penicillin, and 100 μg/mL streptomycin at 37 °C/5% CO_2_. Only passages 2–4 underwent analysis, with endothelial identity confirmed by flow cytometry (>95% CD31^+^/VE-cadherin^+^; CST 77699 and 2500). For barrier disruption, confluent monolayers received *Escherichia coli* O111:B4 LPS (Sigma-Aldrich L4391, 1 μg/mL) in 2% FBS medium for the durations specified below. MLMECs were stimulated with LPS for 24 h.

### Quantitative real-time PCR

We extracted total RNA from snap-frozen lung tissue or MLMECs with TRIzol (Invitrogen) and reverse-transcribed 1 μg using the High-Capacity cDNA Reverse Transcription Kit (Applied Biosystems). SYBR Green qPCR (Power SYBR Green PCR Master Mix, Applied Biosystems) on a QuantStudio 6 Flex system was performed using PrimerBank-designed primers for mouse *Gpr84*, *Ocln*, *Yy1*, and *Gapdh* (reference). Cycling entailed 95 °C for 10 min, then 40 cycles of 95 °C for 15 s and 60 °C for 1 min. We quantified relative expression via the 2^−ΔΔCT^ method against *Gapdh*.

### Western blotting

We homogenized frozen lung tissue (30–40 mg) or cultured cells in RIPA buffer containing Roche protease/phosphatase inhibitors, centrifuged lysates at 14,000 × g for 15 min at 4 °C, and quantified protein via BCA (Pierce). After loading 25–40 μg per lane onto 8%–12% SDS-PAGE gels, we transferred proteins to PVDF membranes (Millipore), blocked them for 1 h at room temperature in 5% non-fat milk or BSA/TBST, and probed overnight at 4 °C with primary antibodies against GPR84 (LSBio LS-C804295, 1:500), Occludin (CST 91131, 1:1000), YY1 (CST 46395, 1:1000), or β-actin (CST 4970, 1:2000). Following TBST washes, we incubated blots with HRP-conjugated goat anti-rabbit IgG (CST 7074, 1:3000) for 1 h at room temperature and visualized signals by ECL on a ChemiDoc system (Bio-Rad). ImageJ (NIH) densitometry normalized each band to its lane-matched β-actin signal, reporting results as fold change *versus* sham or vehicle.

### Immunohistochemistry

We fixed lungs via intratracheal instillation of 4% paraformaldehyde at 20 cmH_2_O pressure, embedded them in paraffin, and sectioned at 5 μm. Following xylene deparaffinization and graded ethanol rehydration, we performed antigen retrieval in citrate buffer (pH 6.0, 10 min), quenched endogenous peroxidase with 0.3% H_2_O_2_/methanol (15 min), and blocked with 5% normal goat serum (30 min). Sections were then incubated overnight at 4 °C with anti-GPR84 (LSBio LS-C804295), followed by biotinylated secondary antibody and streptavidin-HRP (Vector Laboratories). Diaminobenzidine served as the chromogen, and haematoxylin as the counterstain. IHC quantification was performed on 5 sections per mouse, with 10 fields analyzed per section. The animal was used as the statistical unit (n = 8 mice per group) to ensure robust biological replication. Integrated optical density was measured in five random 400× fields per section using ImageJ.

### Lung histopathology and injury scoring

A pathologist blinded to group allocation scored H&E-stained 5-μm lung sections for alveolar septal thickening, airspace consolidation, interstitial neutrophil infiltration, proteinaceous debris, and perivascular edema (each 0–4), yielding a composite injury score (maximum 20).

### Pulmonary function measurements

We assessed airway mechanics at designated intervals post-CLP using a flexiVent system (SCIREQ). Under anesthesia, a 20-gauge tracheostomy cannula allowed mechanical ventilation (10 mL/kg tidal volume, 150 breaths/min). The single-compartment model applied to pressure-flow loops generated PEF (mL/s), Raw (cmH_2_O·s/mL), and Cdyn (mL/cmH_2_O); we averaged three consecutive measurements per animal.

### Bronchoalveolar lavage fluid collection and analysis

At 24 h post-CLP, we euthanized mice with pentobarbital overdose, cannulated the trachea, and lavaged lungs four times with 0.8 mL ice-cold PBS/0.5 mM EDTA. After centrifuging pooled BALF (400 × g, 10 min, 4 °C), we stored supernatants at −80 °C for cytokine (TNF-α, IL-6, CXCL15; R&D Systems ELISA) and protein (BCA) quantification. We resuspended cell pellets in 0.2 mL PBS for total leukocyte counts (hemocytometer) and Diff-Quik (Siemens) cytospin differentials (300 cells/slide classified morphologically).

### Evans Blue dye extravasation assay

We quantified pulmonary microvascular permeability via Evans Blue-albumin leakage. At 22 h post-CLP, mice received Evans Blue (30 mg/kg, Sigma-Aldrich E2129) intravenously; 2 h later, we perfused the pulmonary circulation with 10 mL PBS via the right ventricle, excised lungs, and incubated them in formamide (1 mL/100 mg tissue) at 60 °C for 18 h. Spectrophotometric readings at 620 nm against an Evans Blue standard curve yielded values as μg dye per gram wet lung tissue or fold change *versus* sham.

### Lung wet-weight to body-weight ratio

Immediately after euthanasia, we excised intact lungs, blotted them lightly, and recorded wet weight (mg). Dividing this by the pre-sacrifice body weight (g) and multiplying by 100 gave the lung wet-weight-to-body-weight percentage.

### *In vitro* endothelial permeability

For paracellular flux assessment, we seeded MLMECs (1 × 10^5^ cells/cm^2^) onto 0.4-μm Transwell inserts (Corning) and cultured them for 5–7 days until TEER plateaued. After experimental treatments, we added FITC-dextran (40 kDa, Sigma-Aldrich, 1 mg/mL) to the upper chamber and collected lower-chamber aliquots at 60 min for fluorescence measurement (490/520 nm). Simultaneously, we tracked TEER with an EVOM^2^ voltohmmeter and STX2 electrodes (World Precision Instruments), subtracting background resistance and reporting values as Ω·cm^2^. We express paracellular permeability as a fold change *versus* vehicle.

### Lentivirus-mediated YY1 overexpression

We packaged third-generation lentivirus by co-transfecting HEK293T cells with pLV-YY1-IRES-Puromycin, psPAX2, and pMD2.G using Lipofectamine 3000 (Invitrogen); empty pLV-IRES-Puromycin served as a control. After collecting supernatants at 48 and 72 h, filtering through a 0.45 μm filter, and concentrating by ultracentrifugation (25,000 rpm, 2 h, 4 °C), we transduced MLMECs at an MOI of 10 with 8 μg/mL polybrene for 12 h. Puromycin selection (2 μg/mL, 72 h) generated stable pools, with YY1 overexpression confirmed by immunoblotting.

### GPR84 siRNA knockdown in MLMECs

MLMECs were transfected with GPR84-specific siRNA (OriGene, ABIN3269331) or control siRNA (OriGene, ABIN2666550) using Lipofectamine RNAiMAX (Invitrogen) according to the manufacturer’s protocol. Briefly, cells at 60%–70% confluence were incubated with siRNA (50 nM) and transfection reagent for 48 h. Knockdown efficiency (>70%) was confirmed by qPCR and Western blotting prior to functional assays. For rescue experiments, GPR84-silenced MLMECs were treated with GLPG1205 (10 μM) in the presence of LPS (1 μg/mL) for 24 h, after which TEER and permeability were assessed.

### Experimental design and statistical analysis

We selected sample sizes (n = 8–10 mice per group for *in vivo* experiments; N = 4–5 independent replicates for *in vitro* assays) determined based on prior laboratory data with an expected effect size of 0.8, α = 0.05, and power = 0.80 using G*Power software. Animals were block-randomized by body weight into treatment groups, and outcome assessors remained blinded to allocation. All data are presented as mean ± SEM. Two-group comparisons were performed using a two-tailed unpaired Student’s t-test. Two-way ANOVA with Tukey’s *post hoc* test was used for multi-factor experiments (e.g., LPS × GLPG1205, LPS × GLPG1205 × YY1 overexpression). Normality was assessed using the Shapiro-Wilk test, and variance homogeneity was confirmed using Levene’s test. Biological replicates (independent experiments or individual animals) were used as the statistical unit; technical replicates within an experiment were averaged before analysis. Statistical evaluations were conducted in GraphPad Prism 9 (GraphPad Software, San Diego, CA, USA), with statistical significance defined as *P* < 0.05.

## Results

### GPR84 upregulation in human septic shock

To explore whether GPR84, a receptor implicated in inflammatory amplification, shows clinical relevance in human severe sepsis, we examined GPR84 expression in an independent human pediatric sepsis cohort (GSE13904). Using the GEO2R web interface, we compared Day 1 septic shock samples (n = 67) against healthy controls (n = 18). Transcriptomic analysis of GSE13904, restricted to Day 1 septic shock (n = 67) and healthy controls (n = 18), confirmed marked GPR84 upregulation (log_2_FC = +11.18, adj. *P* = 7.75 × 10^−15^; unadjusted *P* = 1.99 × 10^−18^) (Figure 1). The magnitude of this effect, while striking, should be interpreted with caution given the inherent heterogeneity of pediatric septic shock samples. This result positions GPR84 as a robust feature of severe human sepsis.

**FIGURE 1:**
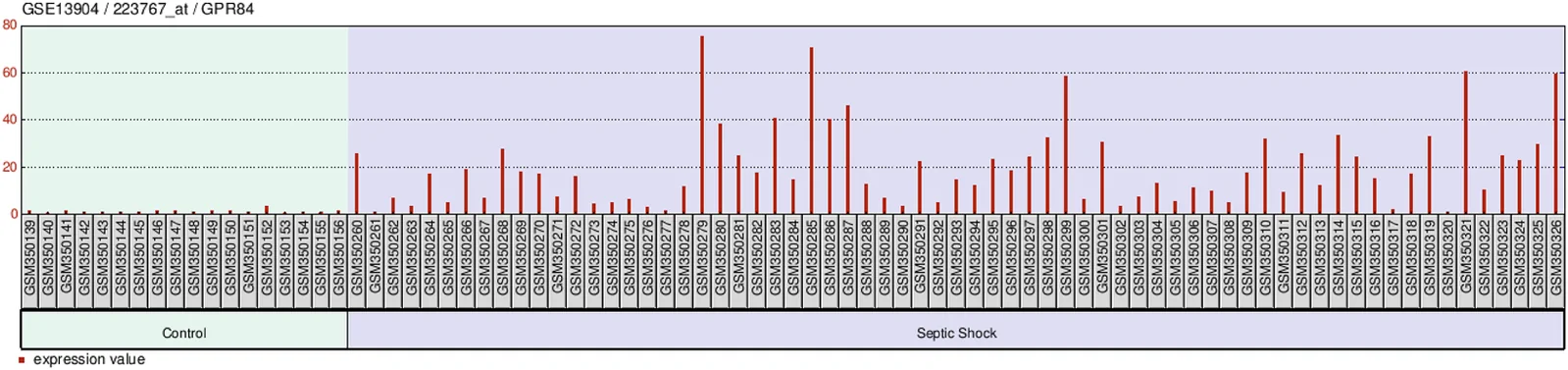
GPR84 induction in human septic shock. GEO2R interrogation of GSE13904. Day 1 pediatric septic shock (n = 67) *versus* healthy controls (n = 18). Probe 223,767_at: log_2_FC = +11.18; adj. *P* = 7.75 × 10^−15^. Profile graph verified the marked GPR84 elevation in shock.

### GPR84 increases in the lung after polymicrobial sepsis

Having confirmed that GPR84 is markedly upregulated in human septic shock, we next examined whether this receptor is similarly induced in the lung during polymicrobial sepsis in mice. We began by asking whether the orphan receptor GPR84 becomes more abundant in the lung during systemic infection. Transcripts coding for GPR84, measured by real-time PCR, were noticeably higher in tissue taken from septic animals than in samples from sham-operated controls (Figure 2A). A comparable rise was found when we examined protein levels. Western blotting of whole-lung extracts showed a clear band intensification in the CLP group (Figure 2B). Immunostaining of tissue sections gave the same picture, with quantitative densitometry confirming stronger reactivity distributed through the alveolar-interstitial compartment of septic mice (Figure 2C). The cellular source of the increased GPR84 immunoreactivity cannot be determined from the present analysis. Separate experiments in cultured MLMECs show that endothelial cells can respond to pharmacological GPR84 inhibition. Elevated expression of GPR84 thus appeared across transcriptional and translational readouts, indicating that polymicrobial sepsis triggers a uniform upregulation of this receptor in pulmonary parenchyma.

**FIGURE 2:**
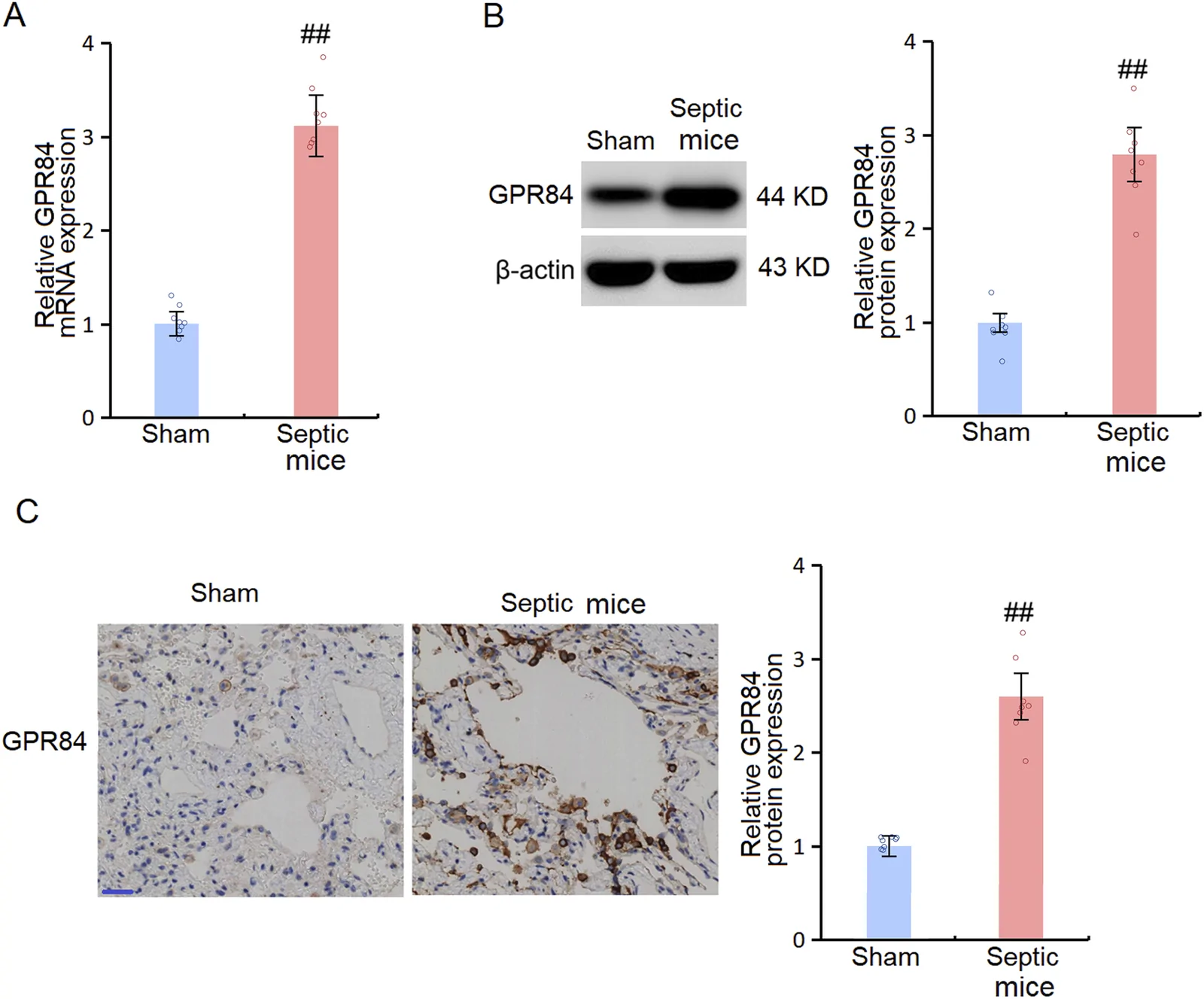
GPR84 is induced in septic mouse lungs. Transcript amounts, measured by real-time PCR, were higher in CLP than in sham animals **(A)**. Immunoblotting showed a corresponding rise in protein **(B)**. Immunohistochemical detection with densitometry confirmed greater receptor abundance in tissue sections. Scale bar, 100 μm **(C)**. n = 8 per group. ##*P* < 0.01 vs. sham.

### Blocking GPR84 lessens histological injury and preserves lung mechanics

Given the induction of GPR84 in the septic lung, we next tested whether its pharmacological blockade would ameliorate the structural and functional abnormalities characteristic of sepsis-associated ALI. Lung sections stained with hematoxylin and eosin from untreated CLP mice displayed thickened septa, interstitial hypercellularity, and patchy airspace filling; injury scores were accordingly much higher than those of sham animals (Figure 3A). Mice given GLPG1205 or TUG-2181 showed far less architectural disruption, with scores considerably closer to sham values. Turning to lung function, peak expiratory flow dropped sharply after CLP but remained significantly higher in both groups that received a GPR84 inhibitor (Figure 3B). Airway resistance, measured as total respiratory system resistance, climbed in the untreated septic cohort and was substantially lower in the GLPG1205-treated and TUG-2181-treated groups (Figure 3C). Dynamic compliance followed an inverse pattern: it decreased markedly after CLP and rose back toward the normal range with GLPG1205 or TUG-2181, both significantly higher than the ALI group (Figure 3D). Both inhibitors gave similar degrees of improvement, making a case that pharmacological GPR84 blockade limits morphologic injury and largely conserves ventilatory performance.

**FIGURE 3:**
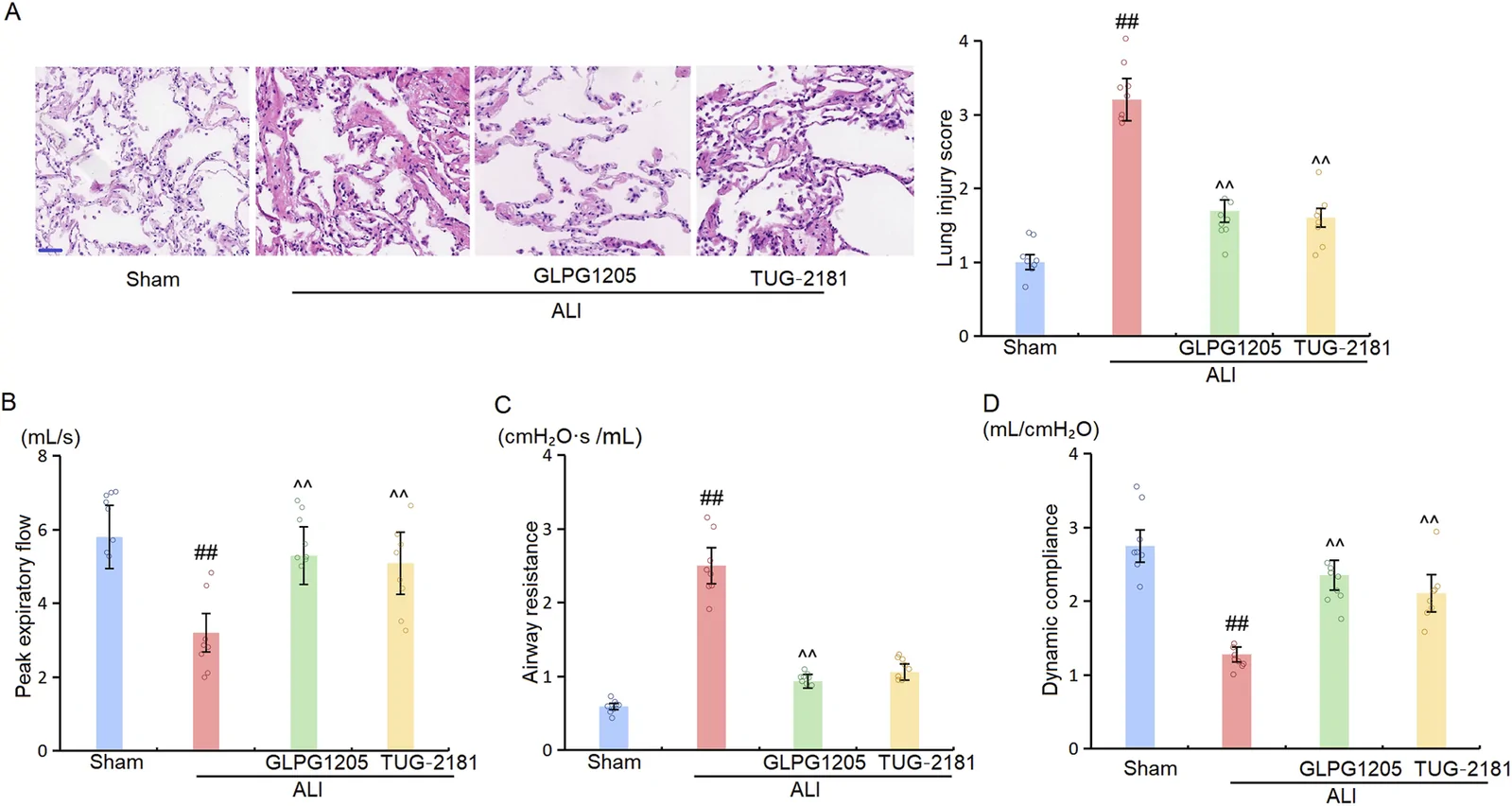
Pharmacological GPR84 blockade reduces histological injury and preserves lung function **(A)** Representative H&E images and lung injury scores. Scale bar, 100 μm **(B)** Peak expiratory flow (PEF) **(C)** Airway resistance (Raw) **(D)** Dynamic compliance (Cdyn) for sham, CLP, CLP + GLPG1205 (10 mg/kg), and CLP + TUG-2181 (20 mg/kg) groups. Data are mean ± SEM (n = 8 per group). ##*P* < 0.01 vs. Sham; ^^*P* < 0.01 vs. CLP.

### Inflammatory indices in airspace fluid are reduced by GPR84 inhibition

We next asked whether the histologic protection conferred by GPR84 inhibition was associated with reduced local airway inflammation. Because excessive cytokine release and leukocyte recruitment drive ALI progression, we next profiled bronchoalveolar lavage fluid. Tumor necrosis factor-α concentrations, which climbed steeply in the untreated septic group, were brought down by GLPG1205 and by TUG-2181 (Figure 4A). Interleukin-6, a cytokine that promotes acute-phase responses and endothelial activation, behaved similarly: it rose after CLP and was partly normalized by either GPR84 inhibitor (Figure 4B). The neutrophil-attracting chemokine CXCL15 was also higher in the ALI cohort and was blunted in the two treatment arms (Figure 4C). Total cell numbers recovered in lavage fluid were greater in septic mice than in sham controls; both inhibitor-treated groups displayed lower total counts (Figure 4D). When we specifically counted neutrophils, their absolute numbers in the alveolar compartment were markedly elevated by CLP and were significantly reduced by GPR84 blockade (Figure 4E). These data show that impeding GPR84 signaling dampens multiple arms of the inflammatory cascade within the airspace.

**FIGURE 4:**
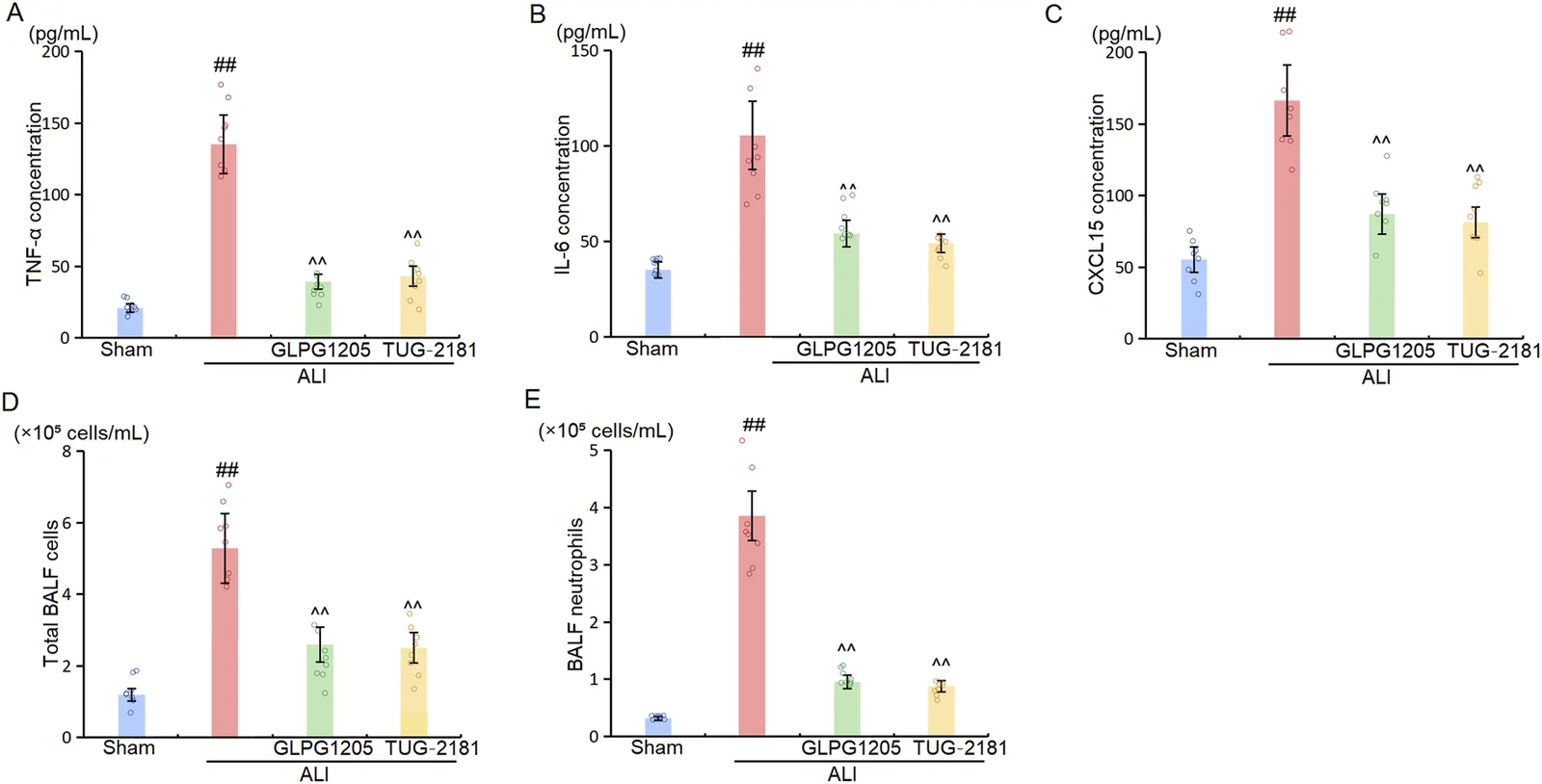
Cytokine levels and leukocyte accumulation in BALF. Concentrations of TNF-α **(A)**, IL-6 **(B)**, and CXCL15 **(C)** dropped with GPR84 inhibition. Total cell counts **(D)** and neutrophil numbers **(E)** followed the same pattern. n = 8. ##*P* < 0.01 vs. sham; ^^*P* < 0.01 vs. CLP.

### Vascular leakage and pulmonary water accumulation are attenuated by receptor blockade

The anti-inflammatory effects observed with GPR84 blockade prompted us to evaluate its impact on the hallmark endothelial barrier dysfunction that underlies pulmonary edema in sepsis. We used three separate readouts to gauge whether GPR84 inhibition restrains permeability. Evans Blue dye extravasation, a marker of albumin leakage into the lung interstitium, nearly doubled in septic mice relative to shams; mice treated with GLPG1205 or TUG-2181 exhibited substantially less dye escape (Figure 5A). Lavage fluid protein content, which reflects loss of size-selective barrier function, rose sharply after CLP and was reduced to a moderate elevation in the two inhibitor groups (Figure 5B). As a gravimetric index of pulmonary water burden, we measured lung wet weight relative to body weight. This ratio was roughly twice as high in the untreated septic group as in sham animals, and much of that difference was eliminated by either compound (Figure 5C). Concordant results across dye leakage, protein transudation, and tissue water content point to a meaningful tightening of the alveolar-capillary barrier when GPR84 is pharmacologically blocked.

**FIGURE 5:**
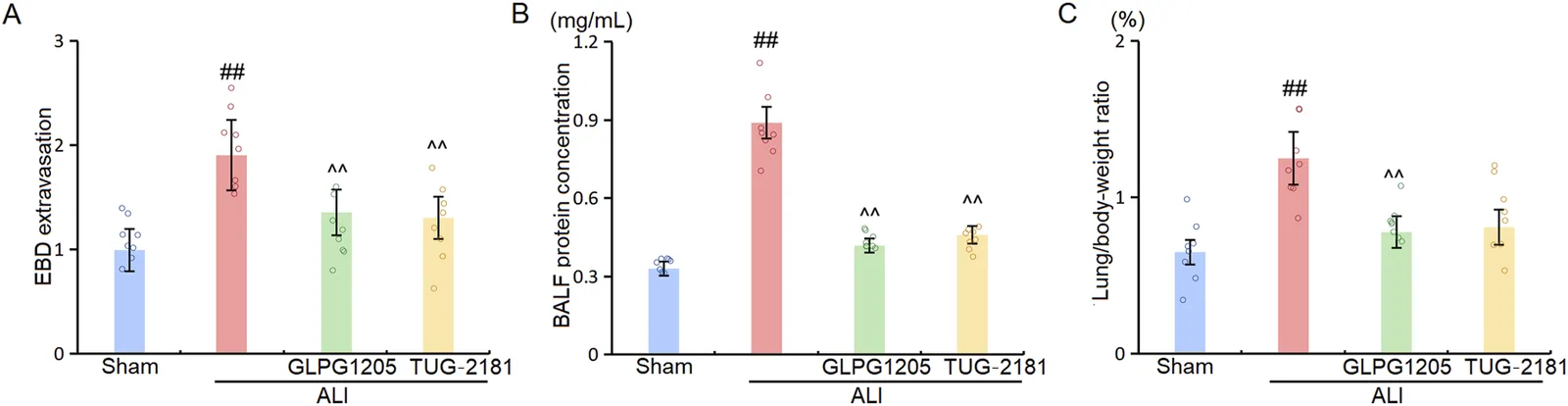
GPR84 inhibition decreases vascular leakage **(A)** Evans blue dye extravasation **(B)** BALF total protein **(C)** Lung wet-weight/body-weight ratio. All three indices were lower in inhibitor-treated septic mice. n = 8. ##*P* < 0.01 vs. sham; ^^*P* < 0.01 vs. CLP.

### Occludin loss in septic lung is counteracted by GPR84 inhibitors

We next investigated whether the functional barrier protection was accompanied by preservation of tight junction proteins, particularly Occludin, in lung tissue. Tight junction strands rely on transmembrane components such as Occludin, and we asked whether the observed barrier improvement was mirrored by changes in this protein. *Ocln* mRNA content, referenced to the sham group, fell in the lungs of untreated septic animals (Figure 6A). The decline was largely reversed by GLPG1205 and to a comparable extent by TUG-2181. Protein immunoblot data were in line with the transcript results: Occludin abundance was decreased in the ALI group, and the drop was substantially corrected by both inhibitors (Figure 6B). The parallel restoration of *Ocln* mRNA and protein implies that GPR84 blockade helps maintain junctional component expression during polymicrobial sepsis, consistent with the functional permeability findings.

**FIGURE 6:**
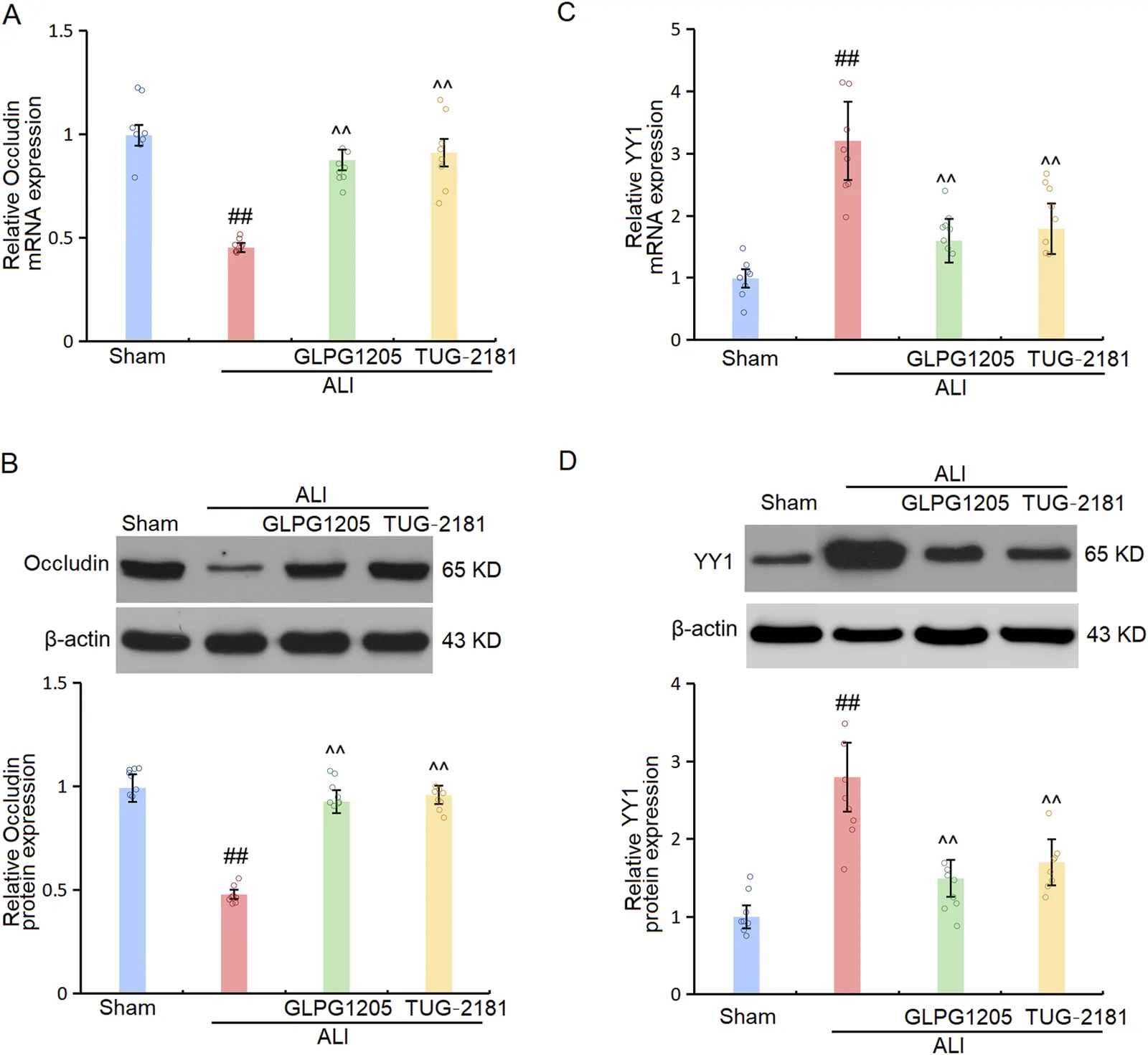
Occludin and YY1 expression in lung tissue **(A)** *Ocln* mRNA and **(B)** Occludin protein abundance fell after CLP and recovered partially with GLPG1205 or TUG-2181 **(C)** YY1 mRNA and **(D)** protein levels were elevated in CLP lungs and partially normalized by GLPG1205 or TUG-2181. n = 8. ##*P* < 0.01 vs. sham; ^^*P* < 0.01 vs. CLP.

### YY1 is induced in septic lung tissue and suppressed by GPR84 inhibition

Given that YY1 is a known transcriptional repressor of Occludin, we examined whether GPR84 inhibition influences YY1 expression in the septic lung. YY1 mRNA and protein levels in lung tissue were assessed following CLP with or without GPR84 inhibitor treatment. YY1 mRNA was elevated 3.20 ± 0.35-fold in CLP lungs (P < 0.01 vs. sham) and was significantly reduced by GLPG1205 (1.60 ± 0.18-fold, P < 0.01 vs. CLP) and TUG-2181 (1.80 ± 0.22-fold, P < 0.01 vs. CLP). Similarly, YY1 protein increased 2.80 ± 0.30-fold after CLP and was partially normalized by GLPG1205 (1.50 ± 0.16-fold) or TUG-2181 (1.70 ± 0.20-fold), both P < 0.01 vs. CLP (Figures 6C,D). Together, these results demonstrate that GPR84 inhibition suppresses YY1 expression in the septic lung at both the transcriptional and protein levels, consistent with the hypothesis that GPR84 acts upstream of YY1 to regulate Occludin expression and endothelial barrier integrity during sepsis.

### GPR84 inhibition protects cultured pulmonary microvascular endothelium from LPS-induced hyperpermeability

To ascertain whether the observed barrier protection *in vivo* is attributable to a direct action on endothelial cells, we turned to an *in vitro* system using cultured lung microvascular endothelial cells. Mouse lung microvascular endothelial cells were stimulated with LPS, and barrier properties were examined. Macromolecular tracer flux rose sharply after LPS stimulation relative to vehicle-treated controls, whereas the co-presence of GLPG1205 or TUG-2181 restrained that increase (Figure 7A). Transendothelial electrical resistance, which reports on ionic permeability, fell in LPS-exposed monolayers and was preserved at higher values when either inhibitor was added (Figure 7B). At the transcript level, *Ocln* mRNA declined after LPS treatment; both compounds kept mRNA levels partially preserved (Figure 7C). Occludin protein followed a similar course: LPS dropped it, and either inhibitor partly prevented that loss (Figure 7D). The consistency among permeability, resistance, and junctional protein abundance supports a direct endothelial action of GPR84 signaling.

**FIGURE 7:**
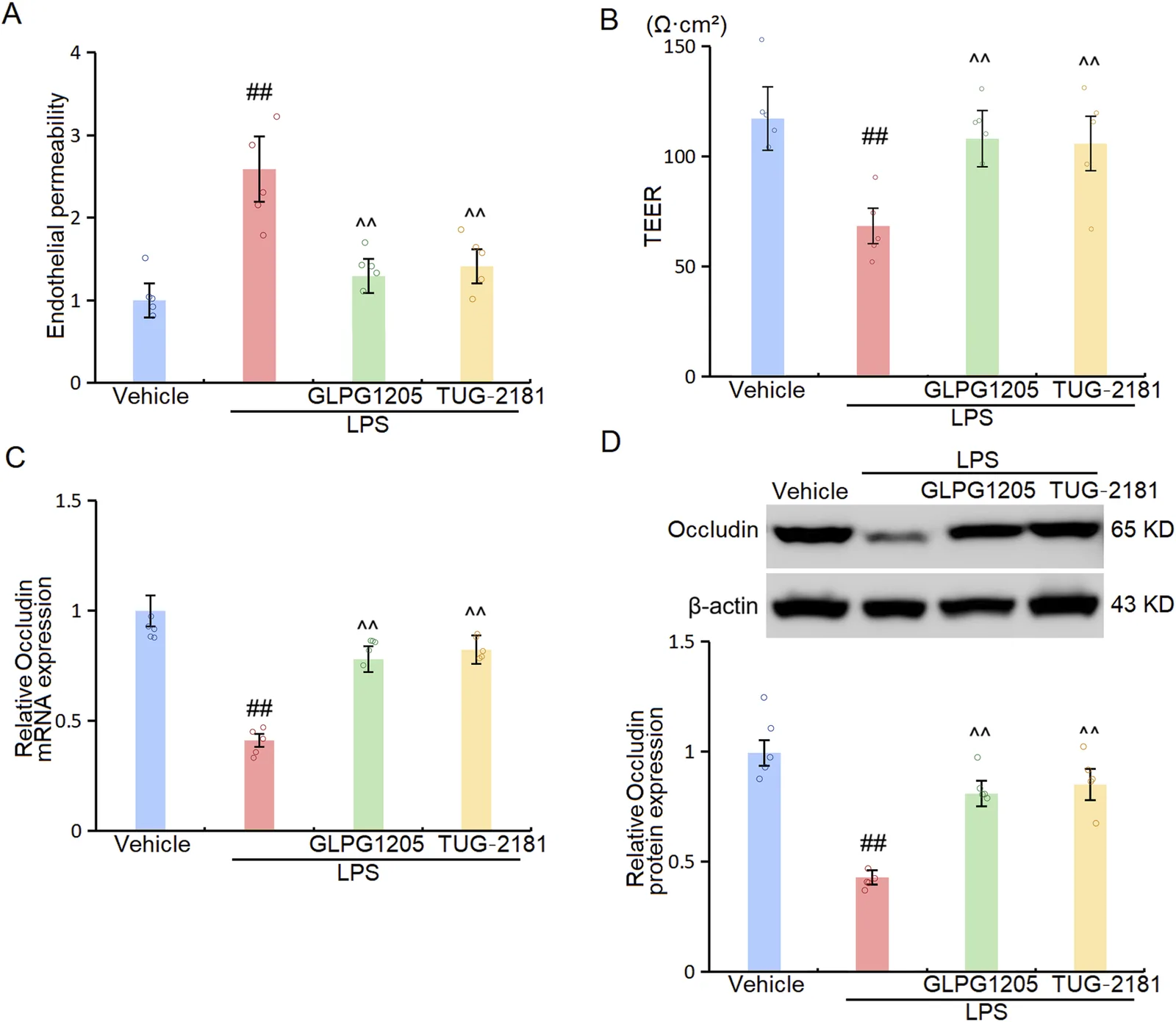
Barrier protection by GPR84 inhibition *in vitro*. LPS-treated MLMECs (stimulated with LPS for 24 h) showed greater FITC-dextran flux **(A)** and lower TEER **(B)**; both changes were blunted by GLPG1205 and TUG-2181. *Ocln* mRNA **(C)** and protein **(D)** mirrored the functional improvement. N = 5 independent experiments. ##*P* < 0.01 vs. vehicle; ^^*P* < 0.01 vs. LPS.

### The transcription factor YY1 is down-regulated by GPR84 blockade in LPS-treated endothelium

We next assessed whether GPR84 blockade modulates YY1 expression directly in endothelial cells by measuring YY1 mRNA and protein levels in LPS-treated MLMECs.Quantitative PCR showed that YY1 mRNA rose markedly in LPS-challenged MLMECs compared with vehicle-treated cells; this induction was reduced by GLPG1205 and likewise by TUG-2181 (Figure 8A). Protein immunoblots matched the transcript pattern: YY1 protein increased with LPS and was lowered to near-baseline levels when either GPR84 inhibitor was present (Figure 8B). The coordinate reduction of YY1 mRNA and protein thus indicates that GPR84 activity is needed for full LPS-driven YY1 expression in these endothelial cells.

**FIGURE 8:**
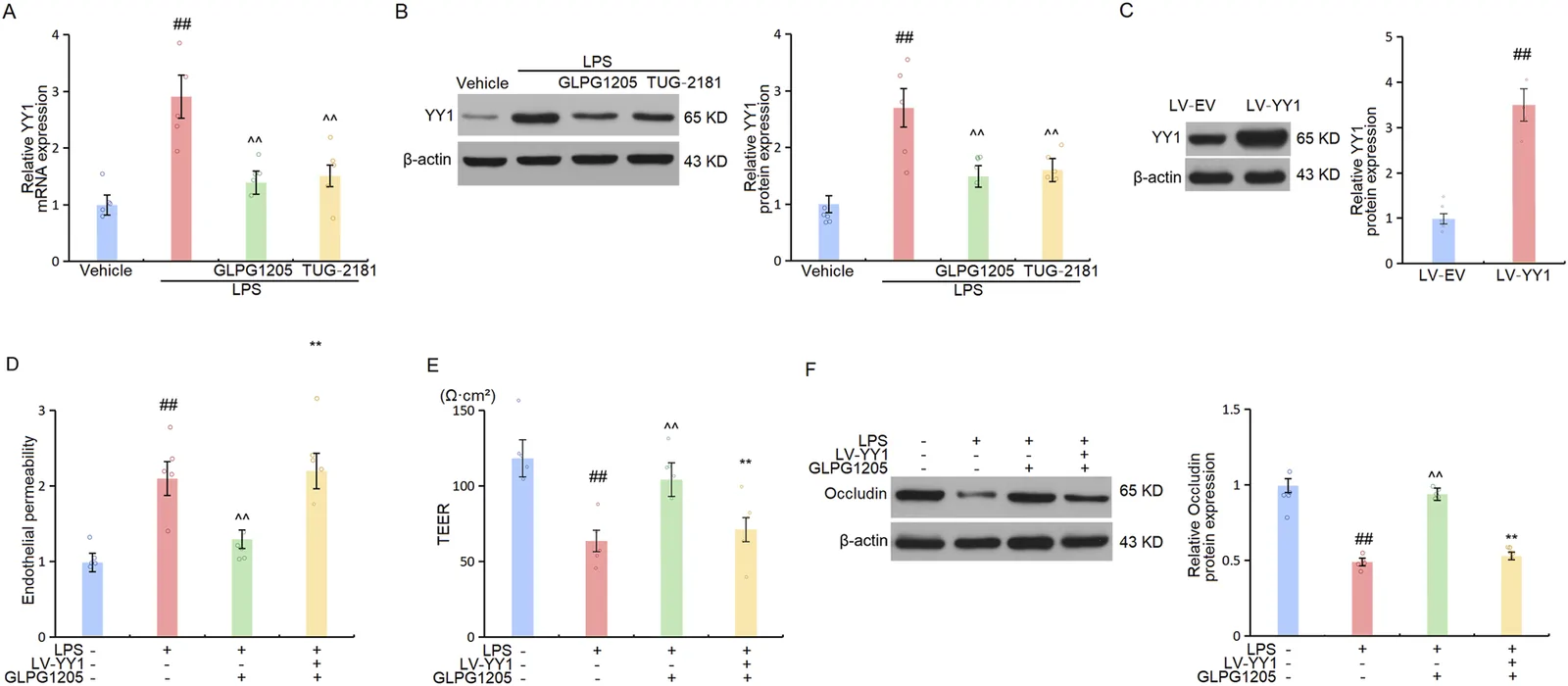
YY1 overexpression reverses the protective effect of GLPG1205 **(A,B)** GLPG1205 and TUG-2181 blunted the LPS-induced increase in YY1 expression **(C)** Confirmation of YY1 overexpression via lentivirus **(D)** Permeability and **(E)** TEER in empty-vector and YY1-transduced MLMECs stimulated with LPS for 24 h, with or without GLPG1205. Maintaining YY1 reduced barrier restoration **(F)** Occludin protein mirrored the functional pattern. N = 5. ##*P* < 0.01 vs. vehicle; ^^*P* < 0.01 vs. LPS; **P < 0.01 vs. LPS + GLPG1205 group.

### YY1 overexpression reduces the barrier protection conferred by GPR84 inhibition

To establish whether YY1 suppression is causally linked to the protective effects of GPR84 inhibition, we overexpressed YY1 in MLMECs and examined the consequences on barrier function. Transduction with a YY1-encoding construct raised the protein several-fold compared with empty-vector controls (Figure 8C). In vector-transduced monolayers, LPS predictably increased permeability, and co-administration of GLPG1205 limited that rise. When YY1 was overexpressed, however, GLPG1205 no longer restrained macromolecular leakage; values moved back toward those observed with LPS alone (Figure 8D). Electrical resistance measurements gave the same answer: barrier recovery seen with GLPG1205 in control cells was largely absent under non-physiological YY1 levels (Figure 8E). Occludin protein showed the same pattern as the permeability and TEER results. GLPG1205 maintained Occludin protein in empty-vector cultures, but this maintenance was lost when YY1 was expressed at supraphysiological levels (Figure 8F). These findings indicate that YY1 suppression contributes to the barrier-protective effect of GPR84 inhibition against LPS- induced barrier breakdown in this *in vitro* model, and suggest that additional downstream mediators may also contribute.

### GPR84 siRNA knockdown phenocopies pharmacological inhibition

To definitively attribute the observed barrier protection to GPR84 and rule out off-target effects of the pharmacological inhibitors, we performed siRNA-mediated knockdown of GPR84 in MLMECs. MLMECs were transfected with GPR84-specific siRNA (OriGene, ABIN3269331), achieving >70% knockdown efficiency (protein: 0.28 ± 0.05-fold; Figure 9A). GPR84-siRNA significantly attenuated LPS-induced TEER reduction (220.1 ± 17.8 Ω cm^2^ vs. 130.5 ± 12.6 Ω cm^2^ in siControl + LPS, P < 0.01) and FITC-dextran permeability (1.40 ± 0.15-fold vs. 3.20 ± 0.30-fold in siControl + LPS, P < 0.01; Figures 9B,C), phenocopying the effects of pharmacological inhibitors. When GPR84 was silenced by siRNA, co-administration of GLPG1205 did not confer additional barrier protection beyond the siRNA effect alone (TEER: 228.8 ± 20.5 Ω cm^2^ vs. 223.6 ± 18.2 Ω cm^2^; Permeability: 1.35 ± 0.16-fold vs. 1.45 ± 0.13-fold; both P = 0.21 and P = 0.23, ns), confirming on-target pharmacology (Figures 9D,E). These findings suggest that the barrier-protective effects of GLPG1205 are mediated through GPR84 in this *in vitro* model, as GLPG1205 conferred no additional protection beyond GPR84 knockdown alone.

**FIGURE 9:**
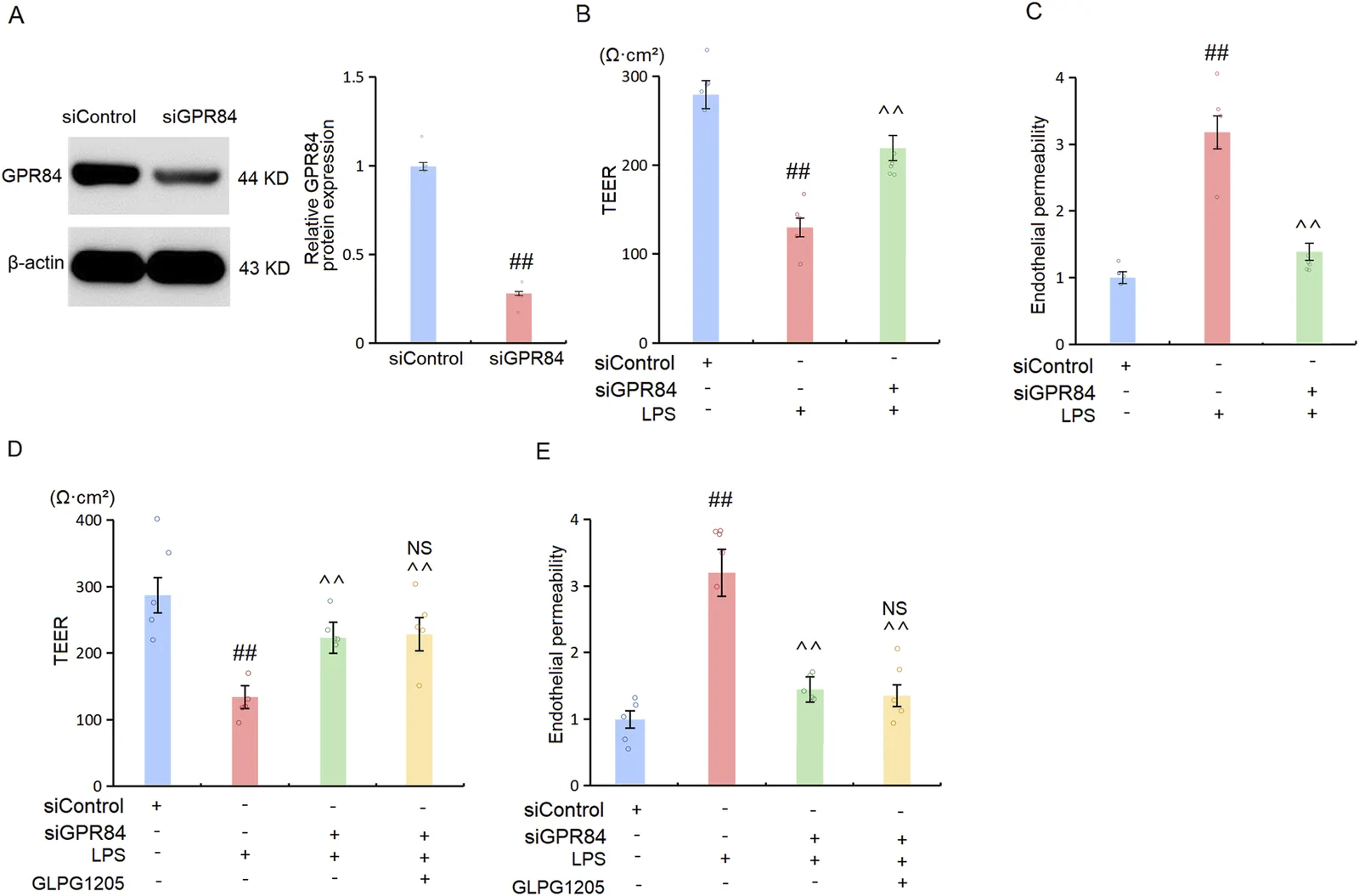
GPR84 siRNA knockdown phenocopies pharmacological inhibition and confirms on-target pharmacology **(A)** GPR84 siRNA knockdown efficiency (>70%) was confirmed by Western blot **(B)** TEER and **(C)** FITC-dextran permeability in siControl- and siGPR84-transduced MLMECs stimulated with LPS for 24 h **(D–E)** When GPR84 was silenced by siRNA, co-administration of GLPG1205 did not confer additional barrier protection beyond the siRNA effect alone, confirming on-target pharmacology. N = 5 independent experiments. ##*P* < 0.01 vs. siControl + Vehicle; ^^*P* < 0.01 vs. siControl + LPS; ns, not significant. Data are presented as mean ± SEM.

## Discussion

Sepsis-associated acute lung injury continues to exact a heavy clinical toll, and the search for druggable targets that directly address vascular integrity has been slow. The present study adds a new dimension to this effort by identifying GPR84 as a receptor whose pharmacological blockade protects the pulmonary endothelial barrier during polymicrobial sepsis, and by implicating YY1 suppression as a downstream event associated with this benefit.

The canonical picture of GPR84 biology has been drawn almost entirely from myeloid cell studies. The receptor is abundant in neutrophils and macrophages, where its engagement by medium-chain fatty acids or synthetic agonists drives chemotaxis, reactive oxygen species generation, degranulation, and the secretion of IL-8 and other mediators (Luscombe et al., 2024; Li et al., 2023). In the context of acute lung inflammation, recent work has shown that GPR84 rises sharply in BALF cells after LPS challenge, and that either genetic deletion or pharmacological blockade reduces neutrophil influx, oxidative markers, and tissue injury (Fredriksson et al., 2022). Our *in vivo* cytokine, lavage cell, and histology data fit comfortably with that earlier work. The drop in TNF-α, IL-6, and CXCL15, together with fewer total leukocytes and neutrophils in the airspaces of inhibitor-treated septic mice, confirms that GPR84 sustains multiple arms of the inflammatory cascade within the lung. Recent studies have further expanded our understanding of the inflammatory microenvironment in sepsis-associated lung injury. Zhang et al. demonstrated that FKBP5 is markedly upregulated in patients with sepsis and correlates with disease severity; mechanistically, FKBP5-mediated necroptosis of alveolar fibroblasts triggers NF-κB activation and pro-inflammatory cytokine release, establishing an inflammatory microenvironment that perpetuates lung injury (Zhang et al., 2026). What our study adds is the demonstration that this attenuation of inflammation is mirrored by a parallel tightening of the vascular barrier—less dye extravasation, lower lavage protein, and less pulmonary water accumulation—suggesting that GPR84 blockade does not simply cool inflammation but actively preserves endothelial competence.

However, whether GPR84 can directly modulate endothelial function has remained a largely open question. The receptor’s transcript has been detected in pulmonary endothelial cells, and one recent report showed that a novel GPR84 inhibitor reduced pyroptotic signaling in the pulmonary vasculature of diabetic mice (Gao et al., 2025). Those data hinted at endothelial relevance but did not examine junctional proteins or paracellular permeability. Our experiments in cultured MLMECs fill that gap. LPS predictably elevated monolayer permeability and depressed TEER, and both GLPG1205 and TUG-2181 restrained those changes. *Ocln* mRNA and protein tracked the functional endpoints, falling with LPS and recovering partially under receptor blockade. That two chemically dissimilar inhibitors produced similar outcomes in a reductionist endothelial system argues strongly for an on-target effect that is intrinsic to the endothelial layer, rather than one that merely reflects attenuated myeloid cell activation. This conclusion is consistent with emerging evidence that several GPCRs expressed in the pulmonary vasculature can be harnessed to limit barrier breakdown (Schulze et al., 2022; Recio et al., 2018). Beyond GPCRs, recent studies have identified additional mechanisms that protect endothelial barrier integrity in lung injury. Zhang et al. demonstrated that heparan sulfate, a key component of the endothelial glycocalyx, acts in synergy with tight junction proteins through STAT3 signaling to maintain the endothelial barrier and prevent LPS-induced lung injury (Zhang et al., 2025).

The identification of YY1 as a downstream transcriptional mediator of GPR84 signaling in endothelial cells is, to our knowledge, a new finding. YY1 is a zinc-finger protein with well-characterized dual activator and repressor activities, and its roles in vascular development, angiogenesis, and vascular normalization are increasingly appreciated (Liu et al., 2020; Wang X. et al., 2023). In the lung endothelium specifically, foundational work established that YY1 binds a distal element of the Occludin promoter and represses transcription, explaining why Occludin is low in lung relative to brain vessels (Zhang et al., 2020). Our data show that LPS drives a roughly three-fold increase in YY1 mRNA and protein in MLMECs, and that GPR84 blockade substantially blunts this induction. More tellingly, the rescue experiment, in which enforced YY1 expression erased the barrier-protective effect of GLPG1205 across permeability, TEER, and Occludin protein endpoints, supports a functional role for YY1 suppression in the endothelial protection that follows GPR84 inhibition. This finding resonates with functional studies of YY1 in microglia, where YY1 overexpression promoted M2 polarization and reduced neuroinflammation in a sepsis-associated encephalopathy model (Peng et al., 2022), illustrating the context-dependent nature of YY1-mediated transcriptional programs in different disease settings, but the direct tie to Occludin regulation in pulmonary endothelium sets our work apart.

The signal transduction pathway connecting GPR84 activation to YY1 induction remains uncharacterized in the context of pulmonary endothelial cells. GPR84 predominantly couples to Gαi/o proteins and can also recruit β-arrestin adaptor proteins, which regulate receptor internalization and desensitization and can scaffold independent downstream signaling cascades (Marsango and Milligan, 2024). In other cell types, GPR84 activation has been shown to stimulate AKT and ERK1/2 kinase cascades (Marsango and Milligan, 2024; Forsman et al., 2024); AKT activation has been shown to induce YY1 expression in lung cells (Guo et al., 2015), and YY1 activity can be modulated by phosphorylation (Zheng et al., 2010), suggesting that kinase cascades downstream of GPR84 could potentially influence YY1-mediated transcription. It is therefore plausible that similar signaling routes operate in LPS-treated pulmonary endothelium, though this remains a hypothesis. Our current data do not identify the specific kinase(s) responsible for the GPR84-to-YY1 link in endothelial cells; future studies employing selective inhibitors of AKT and ERK1/2, combined with YY1 phosphorylation profiling, should aim to delineate this pathway.

Several limitations of this study warrant consideration. First, although our data suggest a correlative link between GPR84 inhibition, YY1 suppression, and Occludin preservation, definitive *in vivo* evidence for a causal YY1-dependent mechanism remains absent. The rescue experiment used YY1 overexpression. Complementary loss-of-function experiments, such as endothelial-specific *Yy1* deletion, would help to test necessity. Endothelial-specific *Yy1* knockout or overexpression, coupled with ChIP-qPCR to quantify YY1 occupancy at the *Ocln* promoter in the CLP model, will be required to validate this axis (Sade et al., 2009; Zhang et al., 2020). Second, GPR84 inhibitors were administered 2 h after CLP induction, representing a prophylactic rather than a therapeutic intervention for established lung injury. Since septic patients typically present after organ dysfunction has developed, delayed treatment protocols (e.g., 6–12 h post-CLP) are needed to more closely approximate clinical practice. Third, although the two chemically distinct inhibitors GLPG1205 and TUG-2181 lower the risk of off-target effects, endothelial-specific Gpr84 knockout mice are still required for definitive *in vivo* genetic validation (Mancini et al., 2019). We partially addressed this gap via siRNA-mediated GPR84 knockdown in MLMECs (>70% efficiency), which alone alleviated LPS-induced barrier disruption; co-administration of GLPG1205 produced no additional protection, confirming on-target pharmacology *in vitro*. Fourth, the detailed signaling cascade connecting GPR84 activation to YY1 upregulation remains uncharacterized. GPR84 signals through Gαi/o and recruits β-arrestin adaptors (Wang et al., 2024), with downstream ERK1/2 and AKT kinases capable of modulating YY1 stability and transcriptional activity; however, which of these cascades predominates in pulmonary endothelial cells awaits further investigation. Fifth, we did not evaluate bacterial loads in blood, peritoneal fluid, or lung tissue, nor did we conduct formal survival analysis to assess whether GPR84 inhibition impairs myeloid-dependent host defense. Although reduced neutrophil infiltration alleviated pulmonary tissue injury in our model, compromised bacterial clearance cannot be ruled out as a potential safety concern. The numerically lower mortality in inhibitor-treated mice (0/10 vs. 2/10 in CLP) hints at a survival benefit; however, the study was not powered for this endpoint, and this observation requires prospective validation. Sixth, our IHC analysis could not discriminate between GPR84 expression on infiltrating leukocytes *versus* resident pulmonary endothelial cells *in vivo*, as GPR84 is highly expressed by myeloid cells. However, our primary cell culture experiments with MLMECs show that endothelial cells are capable of expressing GPR84 and responding directly to its pharmacological inhibition, indicating that endothelial cells can respond to GPR84 inhibition *in vitro*, although the *in vivo* cellular source remains unresolved. Seventh, all experiments relied exclusively on young male C57BL/6J mice. Given well-documented sex differences in sepsis immune responses, our findings cannot be directly extrapolated to female animals or other genetic backgrounds. Eighth, the CLP polymicrobial sepsis model cannot fully recapitulate the broad heterogeneity of human sepsis, which inherently limits direct clinical translation of our preclinical findings.

Notwithstanding these caveats, the work carries several implications. It identifies GPR84 antagonism as a pharmacological strategy that simultaneously addresses inflammation and endothelial leak, two processes that reinforce each other in the septic lung. The demonstration that YY1 serves as a functional link between a surface GPCR and tight junction gene repression opens the door to dissecting additional GPCR-YY1 axes that may operate in other vascular beds or in chronic inflammatory conditions. The availability of GLPG1205, a compound that has already been tested in phase II trials for pulmonary fibrosis (Strambu et al., 2023), provides a translational bridge that could accelerate the evaluation of GPR84 blockade in sepsis-associated ARDS, although careful attention to dosing timing and safety in infected patients will be needed.

Future work should extend these observations in several directions. Generating mice with inducible, endothelial-specific deletion of GPR84 would permit a clean test of the relative contributions of myeloid and endothelial GPR84 to ALI. Profiling YY1 protein, its post-translational modifications, and its occupancy of the Occludin promoter in lung endothelium isolated from septic mice treated with or without GPR84 inhibitors would directly bridge the *in vitro-to-in vivo* gap. Delayed treatment protocols, in which inhibitors are administered 6 or 12 h after CLP, would better assess clinical applicability. Finally, screening for the kinase intermediates that relay GPR84 activation to YY1 upregulation could reveal additional druggable nodes in this pathway.

## Conclusion

Blocking GPR84 with GLPG1205 or TUG 2181 reduced lung damage, airspace cytokine levels, and vascular leak in septic mice while restoring Occludin abundance. *In vivo*, this protection was associated with reduced YY1 expression. In cultured endothelial cells, GPR84 inhibition protected against LPS-driven hyperpermeability, and this protection was substantially diminished by YY1 overexpression. These findings link GPR84 to pulmonary inflammation *in vivo* and endothelial barrier protection *in vitro*, the latter involving YY1 and Occludin. Whether this endothelial pathway operates identically in the septic lung requires further investigation. GPR84 inhibition remains a promising candidate therapeutic target for sepsis-associated ALI, warranting further validation.

## Data Availability

The raw data supporting the conclusions of this article will be made available by the authors, without undue reservation.
